# Characterization of the transcriptionally active form of dephosphorylated DctD complexed with dephospho-IIA^Glc^

**DOI:** 10.1128/mbio.00330-24

**Published:** 2024-04-02

**Authors:** Sebin Kang, Bo-Ram Jang, Kyu-Ho Lee

**Affiliations:** 1 Department of Life Science, Sogang University, Seoul, South Korea; The Pennsylvania State University, University Park, Pennsylvania, USA

**Keywords:** bacterial enhancer-binding protein, dephospho-DctD/dephospho-IIA^Glc^complex, dodecameric conformation, upstream activator sequences

## Abstract

Bacterial enhancer-binding proteins (bEBPs) acquire a transcriptionally active state via phosphorylation. However, transcriptional activation by the dephosphorylated form of bEBP has been observed in DctD, which belongs to Group I bEBP. The formation of a complex between dephosphorylated DctD (d-DctD) and dephosphorylated IIA^Glc^ (d-IIA^Glc^) is a prerequisite for the transcriptional activity of d-DctD. In the present study, characteristics of the transcriptionally active complex composed of d-IIA^Glc^ and phosphorylation-deficient DctD (DctD_D57Q_) of *Vibrio vulnificus* were investigated in its multimeric conformation and DNA-binding ability. DctD_D57Q_ formed a homodimer that could not bind to the DNA. In contrast, when DctD_D57Q_ formed a complex with d-IIA^Glc^ in a 1:1 molar ratio, it produced two conformations: dimer and dodecamer of the complex. Only the dodecameric complex exhibited ATP-hydrolyzing activity and DNA-binding affinity. For successful DNA-binding and transcriptional activation by the dodecameric d-IIA^Glc^/DctD_D57Q_ complex, extended upstream activator sequences were required, which encompass the nucleotide sequences homologous to the known DctD-binding site and additional nucleotides downstream. This is the first report to demonstrate the molecular characteristics of a dephosphorylated bEBP complexed with another protein to form a transcriptionally active dodecameric complex, which has an affinity for a specific DNA-binding sequence.

**IMPORTANCE:**

Response regulators belonging to the bacterial two-component regulatory system activate the transcription initiation of their regulons when they are phosphorylated by cognate sensor kinases and oligomerized to the appropriate multimeric states. Recently, it has been shown that a dephosphorylated response regulator, DctD, could activate transcription in a phosphorylation-independent manner in *Vibrio vulnificus*. The dephosphorylated DctD activated transcription as efficiently as phosphorylated DctD when it formed a complex with dephosphorylated form of IIA^Glc^, a component of the glucose-phosphotransferase system. Functional mimicry of this complex with the typical form of transcriptionally active phosphorylated DctD led us to study the molecular characteristics of this heterodimeric complex. Through systematic analyses, it was surprisingly determined that a multimer constituted with 12 complexes gained the ability to hydrolyze ATP and recognize specific upstream activator sequences containing a typical inverted-repeat sequence flanked by distinct nucleotides.

## INTRODUCTION

Transcription factors capable of binding to specific DNA sequences located in the upstream regions, the so-called upstream activator sequences (UASs) (1), of target genes have been named bacterial enhancer-binding proteins (bEBPs) (2). Typically, members of Group I bEBPs must be phosphorylated via a cognate sensor kinase-mediated phosphorelay and oligomerized into appropriate multimeric forms exhibiting ATPase activity to successfully become transcriptional activators (3). DctD, a member of Group I bEBPs, has been initially isolated as the main regulator for the expression of the dicarboxylic acid transporter (DctA) in a nitrogen-fixing *Rhizobium* species (4). Its sensor kinase DctB, comprising a two-component system (TCS) with the response regulator DctD, senses ambient dicarboxylic acids and phosphorylates DctD, thereby activating the transcription of *dctA* genes (5).

DctBD systems have also been found in many bacterial species, including the model foodborne pathogen *Vibrio vulnificus*, in which RpoN-initiated transcription of two distinct gene clusters for exopolysaccharides (EPS) biosynthesis, EPS-II and EPS-III clusters, is activated by DctBD (6). These findings demonstrate that the regulatory roles of DctD may extend beyond the expression of genes related to the uptake and metabolism of dicarboxylic acids. Furthermore, *V. vulnificus* DctD has been shown to exhibit its *in-vivo* transcriptional activity even in the absence of DctB ligands such as dicarboxylic acids. The dephosphorylated form of *V. vulnificus* DctD (d-DctD) transitions into the transcriptionally active state under conditions in which the cellular levels of the dephosphorylated form of the glucose-specific enzyme IIA (d-IIA^Glc^) are sufficient to interact with d-DctD (7). In this novel regulatory pathway, the phosphorelay-independent activation of DctD occurs through the direct interaction of d-DctD with d-IIA^Glc^, which forms a transcriptionally active complex of [d-IIA^Glc^/d-DctD].

Based upon numerous reports regarding the transcriptionally active forms of Group I bEBPs (8), it has been speculated that the complex of [d-IIA^Glc^/d-DctD] forms multimeric state(s) to bind to the UAS and activate the transcription of EPS-II and EPS-III clusters. Therefore, in the present study, we examined the molecular characteristics of the complex consisting of d-DctD and d-IIA^Glc^. Among the multimeric states of the [d-IIA^Glc^/d-DctD] complex, a multimer exhibiting both DNA-binding affinity and ATPase activity was identified. Next, the DNA regions comprising the UAS specifically required for multimeric [d-IIA^Glc^/d-DctD] were localized, and the consensus binding sequences for multimeric [d-IIA^Glc^/d-DctD] were proposed.

## RESULTS

### A phosphorylation-deficient DctD (DctD_D57Q_) forms a transcriptionally inactive dimer ([DctD_D57Q_]_2_)

**Fig 1:**
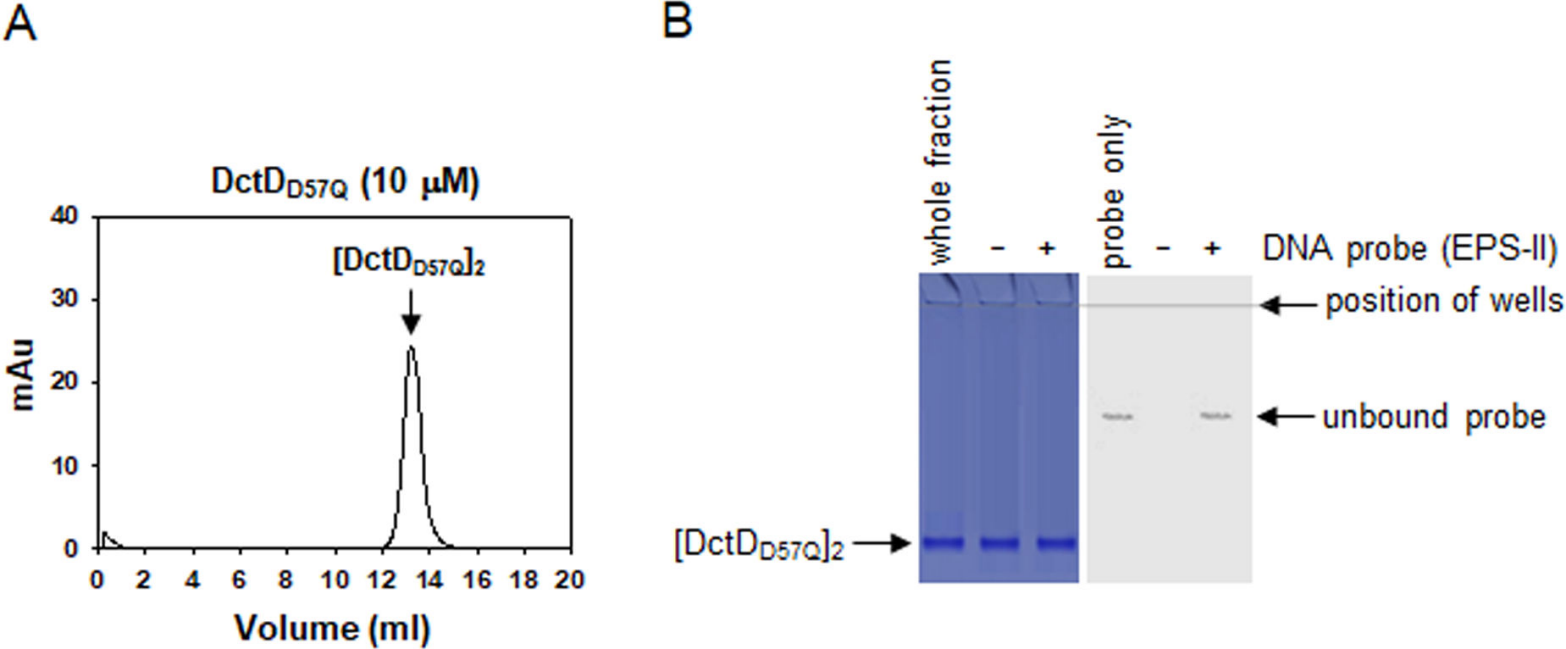
Characterization of the multimeric conformation of DctD_D57Q_ (**A**) Chromatographic profile of DctD_D57Q_. Recombinant protein of DctD_D57Q_ (7), whose calculated molecular weight is 53.53 kDa, was subjected to GPC as described in the Materials and Methods section. The resultant profile showing a single peak at 13.4 mL was presented with the absorbance at 280 nm (mAu). Its size was determined using a regression equation of the standard proteins, as shown in Fig. S1. The fractions (13 and 14 mL) containing this peak were collected and used for electrophoretic mobility shift assay (EMSA) in Fig. 1B. (**B**) DNA-binding characteristic of the dimeric DctD_D57Q_. Approximately 130 nM of the labeled probe from −418 to +62 relative to the transcription initiation site 1 of the EPS-II cluster (6) was incubated with 250 nM dimeric DctD_D57Q_. Reaction mixtures were run on two identical 8% native polyacrylamide gels; one was stained with Coomassie Blue to localize the proteins (left panel), and the other was observed under a phosphoimager to localize the DNA probes (right panel). The first lanes in each gel were run with the whole fraction of DctD_D57Q_ preparation (left panel) and the labeled probe only (right panel). The second and third lanes in each gel were run with the mixtures containing [DctD_D57Q_]_2_ in the absence (−) and presence (+) of DNA probe, respectively.

NtrC, one of the representative members of response regulators belonging to Group I of bEBPs, forms a dimer when not phosphorylated, and this dimer possesses DNA-binding ability (9). However, the dephosphorylated form of DctD (d-DctD) has been shown to be unable to bind to the UASs of targets such as EPS-II and EPS-III clusters in *V. vulnificus* (7). Therefore, we examined the oligomeric state of d-DctD using a phosphorylation-deficient mutant DctD_D57Q_. On gel permeation chromatography (GPC), recombinant DctD_D57Q_ protein produced a major peak at 13.4 mL in its elution profile (Fig. 1A). When this peak volume was extrapolated to a regression equation derived from standard protein markers (Fig. S1) (10), it was converted to a molecular weight (MW) of 117.1 kDa, which approximated the calculated size of the dimeric DctD_D57Q_ (107.1 kDa). To verify that this dimer did not have DNA-binding ability, as previously shown using the whole fraction of DctD_D57Q_ (7), the fractionated dimeric DctD_D57Q_ ([DctD_D57Q_]_2_) was added to a DNA fragment covering −418 to +62 relative to the RpoN-dependent transcription initiation site (TIS, 6) of the EPS-II cluster. The reaction mixtures were run on two identical gels: one was used to localize the proteins by staining with Coomassie Blue, and the other was used to localize the labeled probes via autoradiography (Fig. 1B). Retarded bands of dimeric DctD_D57Q_ and the DNA probe were not observed on either gel, indicating that [DctD_D57Q_]_2_ had no DNA-binding ability.

### DctD_D57Q_ complexed with d-IIA^Glc^ (d-IIA^Glc^/DctD_D57Q_ complex) forms a dimer ([d-IIA^Glc^/DctD_D57Q_]_2_) or a dodecamer ([d-IIA^Glc^/DctD_D57Q_]_12_)

**Fig 2:**
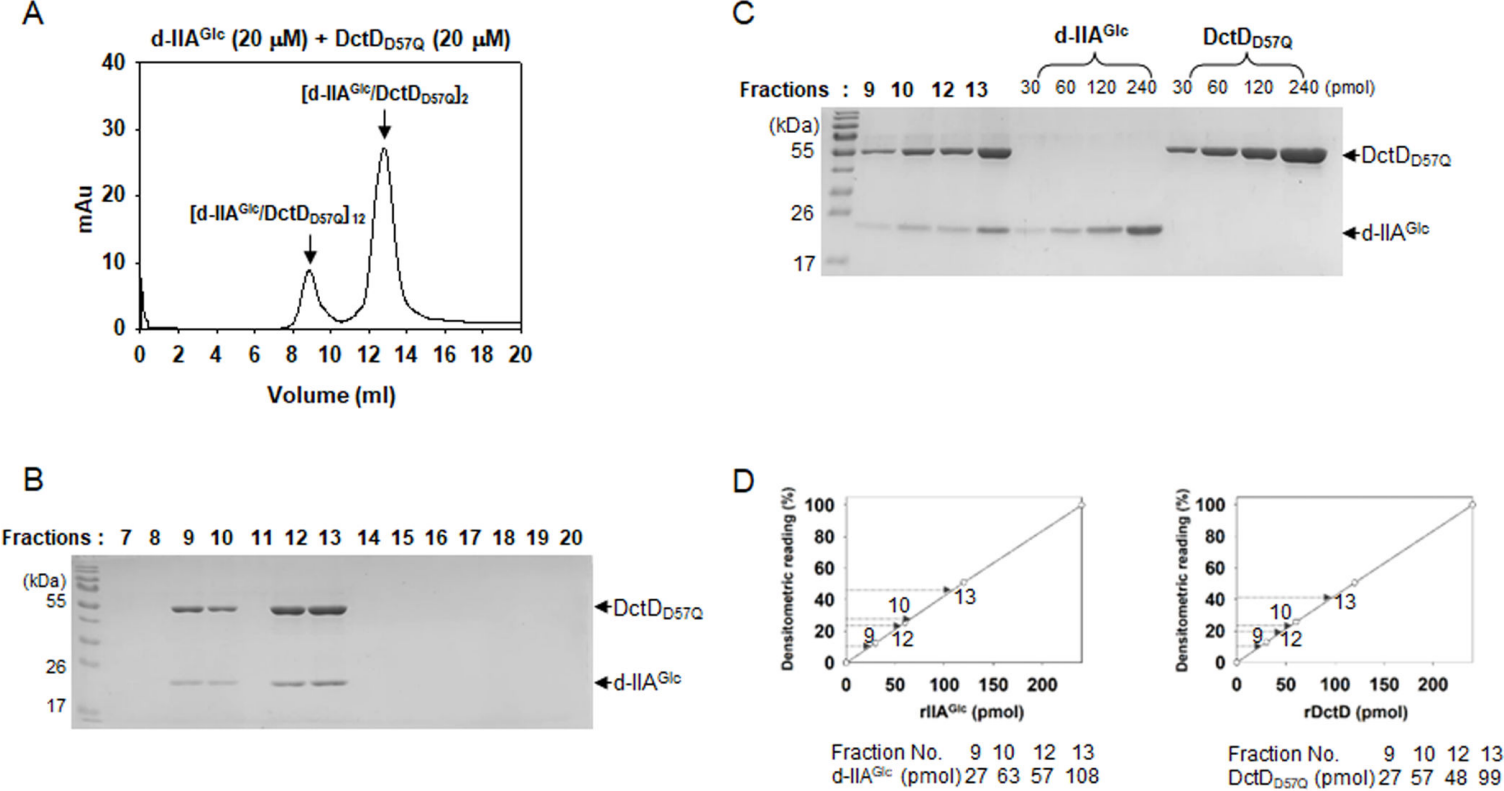
Characterization of the multimeric conformation of the complex composed of dephospho-IIA^Glc^ and DctD_D57Q_ (d-IIA^Glc^/DctD_D57Q_ complex). (**A and B**) Chromatographic and electrophoretic analyses of d-IIA^Glc^/DctD_D57Q_ complex. A mixture of the same concentrations of two recombinant proteins (20 µM each), d-IIA^Glc^ and DctD_D57Q_ (7), was subjected to GPC (**A**), as described in Fig. 1A. Each fraction for the peaks at 8.9 and 12.8 mL was run in an SDS-polyacrylamide gel (**B**). (**C and D**) Compositional analysis of the d-IIA^Glc^/DctD_D57Q_ complex in the two peaks. The fractions for the peaks at 8.9 mL (fractions 9 and 10) and 12.8 mL (fractions 12 and 13) were separated in an SDS-polyacrylamide gel (**C**). For quantitative analysis, the known amounts (30–240 pmol) of each recombinant protein were included in the same gel. Densitometric readings of each band were plotted against the protein amounts to produce the standard curves (D, open circles). Using the standard curves for d-IIA^Glc^ (left graph) and DctD_D57Q_ (right graph), the amounts of each protein in the four fractions were extrapolated and the resultant values were provided below the graphs.

Although DctD_D57Q_ is present in a transcriptionally inactive state, it acquires DNA-binding ability in the presence of dephosphorylated IIA^Glc^ (d-IIA^Glc^), forming a transcriptionally active complex (7). In this study, we analyzed the molecular characteristics of the interaction between the two proteins, such as the multimeric state(s) of the d-IIA^Glc^/DctD_D57Q_ complex and the molar ratio(s) of each protein in the complex. A mixture containing the same concentration (20 µM) of the two recombinant proteins, DctD_D57Q_ and d-IIA^Glc^, was analyzed by GPC. Resultant elution profile showed two distinct peaks at 8.9 and 12.8 mL (Fig. 2A), which were converted to molecular weights of 905.5 and 151.0 kDa, respectively, by extrapolating each peak volume to a regression equation shown in Fig. S1. These converted values approximated the calculated sizes of the dodecamer (902.6 kDa) and dimer (150.4 kDa) of the d-IIA^Glc^/DctD_D57Q_ complex. To verify the two multimers’ MWs determined via GPC analysis, the same mixture was analyzed by an independent method: a gradient gel electrophoresis alongside the two standards, one covering from 180 to 1,800 kDa and the other covering from 66 to 1,048 kDa (Fig. S2). The mixture was resolved into two bands: a higher MW band at 900 kDa and a lower MW band between 180 and 146 kDa. This observation strongly supported the GPC-mediated estimation of the d-IIA^Glc^/DctD_D57Q_ complexes. Subsequent analysis of each fraction collected every 1 mL revealed that the protein bands corresponding to the recombinant proteins of DctD_D57Q_ and IIA^Glc^ appeared only in the lanes of a gel run by the fractions covering the two peaks: fractions 9–10 for a dodecamer and fractions 12–13 for a dimer (Fig. 2B). To further examine the compositional characteristics of each complex, fractions 9, 10, 12, and 13 were separated on an SDS-polyacrylamide gel (Fig. 2C). Using densitometric readings of known amounts of each recombinant protein, ranging from 30 to 240 pmol, which were run in the same gel, the standard curves for DctD_D57Q_ (right panel) and IIA^Glc^ (left panel) were plotted (Fig. 2D). The amounts of DctD_D57Q_ and IIA^Glc^ in the four fractions were estimated, indicating that almost the same ratio of the two proteins was present in each fraction. Fractions 9, 10, 12, and 13 contained 27, 63, 57, and 108 pmol of IIA^Glc^ and 27, 57, 48, and 99 pmol of DctD_D57Q_, respectively.

**Fig 3:**
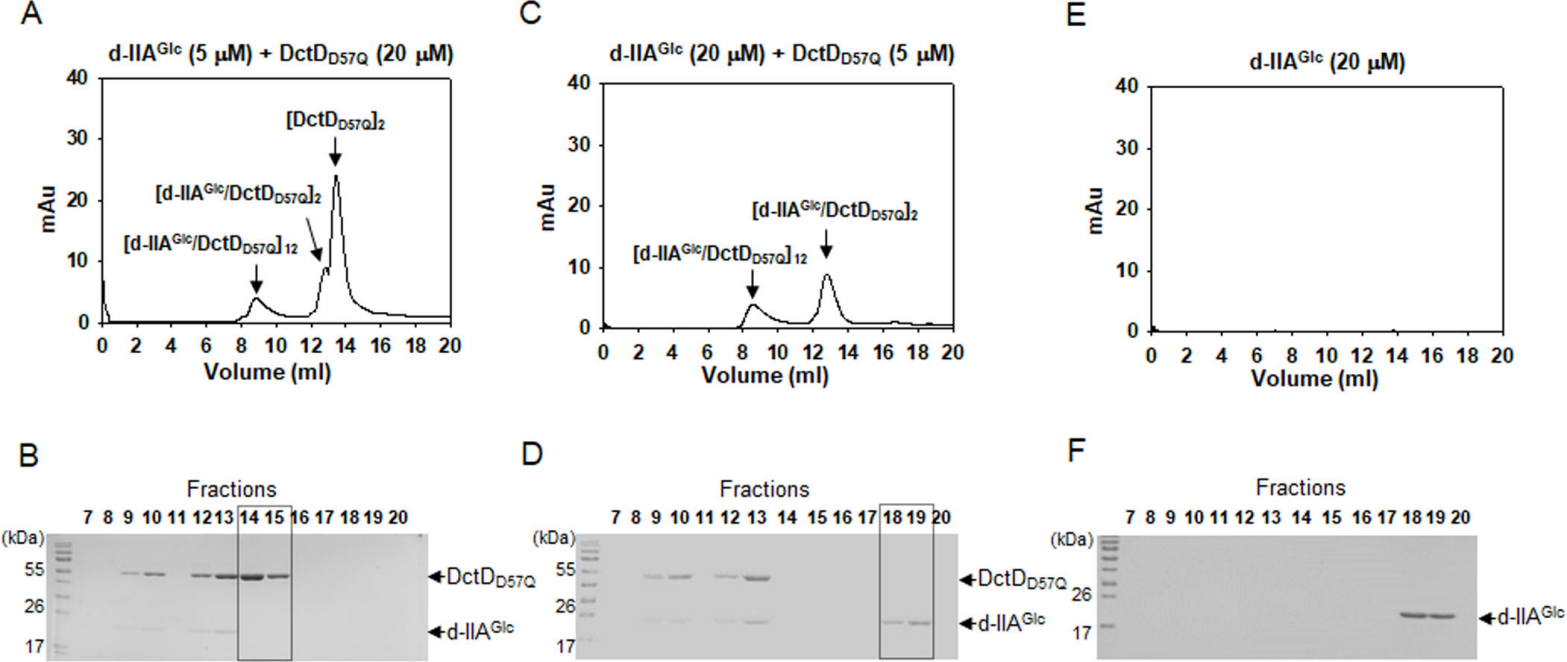
Verification of the 1:1 binding ratio of IIA^Glc^ and DctD in the d-IIA^Glc^/DctD_D57Q_ complexes. Mixtures containing 5 µM d-IIA^Glc^ and 20 µM DctD_D57Q_ (**A and B**) or 20 µM d-IIA^Glc^ and 5 µM DctD_D57Q_ (**C and D**) were analyzed as described in Fig. 2A and B. Both chromatographic and electrophoretic profiles showed the presence of both proteins in the fractions 9, 10, 12, and 13 mL, as shown in Fig. 2. An extra peak corresponding to dimeric DctD_D57Q_ (**A**) was formed in a mixture of 5 µM d-IIA^Glc^ and 20 µM DctD_D57Q_, which was evidenced in an SDS-polyacrylamide gel (B, the fractions 14 and 15 mL were marked with a dashed box). SDS-PAGE analysis of a mixture of 20 µM d-IIA^Glc^ and 5 µM DctD_D57Q_ showed that the fractions 18 and 19 mL contained a protein with the MW of IIA^Glc^ (D, marked with a dashed box). Its identity was verified by profiling d-IIA^Glc^ (20 µM) using chromatographic and electrophoretic analyses (**E and F**).

To confirm the 1:1 molar ratio of the two proteins in either the dimeric or dodecameric form of the d-IIA^Glc^/DctD_D57Q_ complex, mixtures containing different ratios of the two proteins were prepared as follows: 5 µM d-IIA^Glc^ + 20 µM DctD_D57Q_ and 20 µM d-IIA^Glc^ +5 µM DctD_D57Q_. Two mixtures were subjected to GPC and subsequent SDS-PAGE, as shown in Fig. 2. The mixture containing more DctD_D57Q_ than d-IIA^Glc^ (5 µM d-IIA^Glc^ + 20 µM DctD_D57Q_) produced an extra peak at 13.4 mL in the GPC profile in addition to two peaks (8.9 and 12.8 mL) whose heights and areas decreased by 2- to 5-fold compared to those shown in Fig. 2A (Fig. 3A). Fractions 14 and 15, which covered the extra peak at 13.4 mL peak, were revealed to contain only DctD_D57Q_ (Fig. 3B). Therefore, the peak at 13.4 mL contained the dimeric DctD_D57Q_ that was not involved in forming the d-IIA^Glc^/DctD_D57Q_ complex. In contrast, the mixture containing more d-IIA^Glc^ than DctD_D57Q_ (20 µM d-IIA^Glc^ + 5 µM DctD_D57Q_) produced two peaks at 8.9 and 12.8 mL, and their heights and areas were almost the same as those in Fig. 3A (Fig. 3C). However, an SDS-polyacrylamide gel run with each elution showed the presence of a protein band with a MW of IIA^Glc^ in fractions 18 and 19 (Fig. 3D). To prove this band was d-IIA^Glc^ that was not involved in forming the d-IIA^Glc^/DctD_D57Q_ complex, recombinant d-IIA^Glc^ (20 µM) was passed through GPC and the collected fractions were subjected to SDS-PAGE analysis. Although d-IIA^Glc^ could not be detected using GPC equipped with an UV detector (Fig. 3E), its presence was evident in the 18th and 19th fractions (Fig. 3F).

### [d-IIA^Glc^/DctD_D57Q_]_12_ has an ability to bind to DNA and hydrolyze ATP

**Fig 4:**
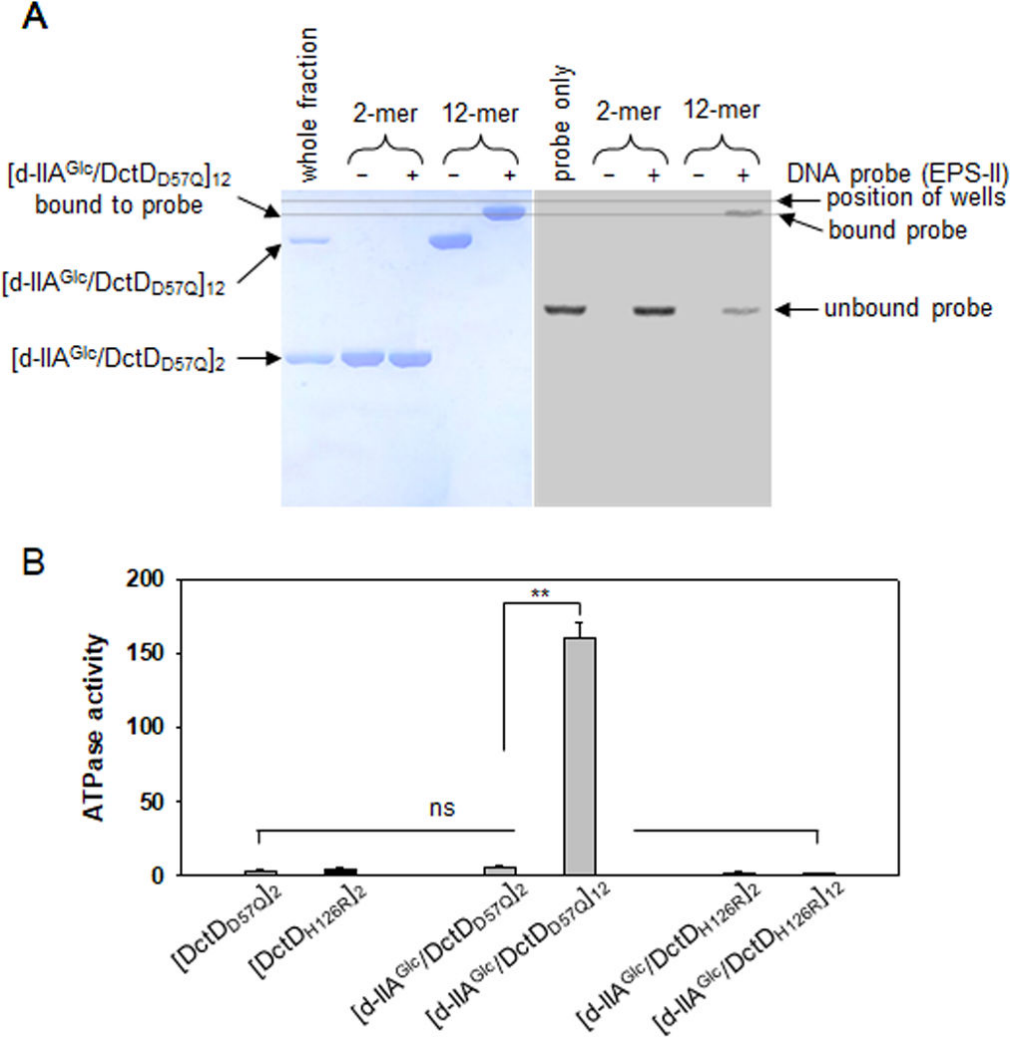
Identification of the transcriptionally active form of d-IIA^Glc^/DctD_D57Q_ complex. (**A**) DNA-binding ability of the multimeric forms of the d-IIA^Glc^/DctD_D57Q_ complex. Approximately 100 nM of a labeled DNA probe used in Fig. 1B was incubated with 2-mer ([d-IIA^Glc^/DctD_D57Q_]_2_) and 12-mer ([d-IIA^Glc^/DctD_D57Q_]_12_) of the complex (500 nM [d-IIA^Glc^/DctD_D57Q_]_1_-equivalents). Reaction mixtures were run on two identical 8% native polyacrylamide gels: one was for localizing the proteins (left panel) and the other was for localizing the DNA probes (right panel). For comparison of the bands corresponding to the d-IIA^Glc^/DctD_D57Q_ complex bound to DNA (left panel) with the probe bound by d-IIA^Glc^/DctD_D57Q_ complex (right panel), a dashed line was drawn parallel to the line positioning the loading wells. The first lanes in each gel were run with the whole fraction of the d-IIA^Glc^/DctD_D57Q_ complex (left panel) and the labeled probe only (right panel). (**B**) ATPase activity of the multimeric forms of the d-IIA^Glc^/DctD_D57Q_ complex. Both [d-IIA^Glc^/DctD_D57Q_]_2_ and [d-IIA^Glc^/DctD_D57Q_]_12_ were fractionated and aliquots containing 2 µM complexes ([d-IIA^Glc^/DctD_D57Q_]_1_-equivalents) were subjected to the ATPase activity assay (11). One micromolar dimeric DctD_D57Q_ ([DctD_D57Q_]_2_) was included in the assay. As a negative control, an ATPase-deficient mutant DctD, DctD_H216R_, was purified as a dephosphorylated state, and then its multimeric forms ([DctD_H216R_]_2_, [d-IIA^Glc^/DctD_H216R_]_2_, and [d-IIA^Glc^/DctD_H216R_]_12_) were added to the ATPase reaction mixture (black bars). The activity was presented as μM phosphate produced by μg of proteins per minute. *P-*values were indicated (**P < 0.001; ns, not significant).

The two multimeric forms, a dimer ([d-IIA^Glc^/DctD_D57Q_]_2_) and a dodecamer ([d-IIA^Glc^/DctD_D57Q_]_12_), of the complex are composed of d-IIA^Glc^ and DctD_D57Q_ in a 1:1 molar ratio. To identify the multimeric form(s) of the complex capable of binding to DNA, the labeled probe shown in Fig. 1 was mixed with [d-IIA^Glc^/DctD_D57Q_]_2_ or [d-IIA^Glc^/DctD_D57Q_]_12_. The mixtures were run on two identical gels, and each gel was stained with Coomassie Blue or exposed to a phosphoimager (Fig. 4A). The mixture containing [d-IIA^Glc^/DctD_D57Q_]_2_ did not show any shifted band on either gel, indicating that the dimeric form of the complex had no DNA-binding ability. In contrast, a mixture containing [d-IIA^Glc^/DctD_D57Q_]_12_ produced retarded bands of protein and DNA, corresponding to a protein band for the probe-bound [d-IIA^Glc^/DctD_D57Q_]_12_ (left panel, Fig. 4A) and a DNA band for the [IIA^Glc^/DctD_D57Q_]_12_-bound probe (right panel, Fig. 4A). This suggests that [IIA^Glc^/DctD_D57Q_]_12_, which exhibits DNA-binding affinity, may be a transcriptionally active form of d-DctD.

It has been reported that transcriptionally active forms of the Group I bEBP have the enzymatic activity to hydrolyze ATP molecules (12). Assays of ATPase activity using [DctD_D57Q_]_2_, [d-IIA^Glc^/DctD_D57Q_]_2_, and [d-IIA^Glc^/DctD_D57Q_]_12_ revealed that only the dodecameric form of the complex exhibited ATPase activity (gray bars, Fig. 4B). To exclude the possibility that the fractions containing [d-IIA^Glc^/DctD_D57Q_]_12_ might have been coeluted with some ATPases, an ATP hydrolysis-deficient DctD (13), DctD_H216R_, was prepared as a dephosphorylated form (d-DctD_H216R_) and used for GPC analysis. The fractions containing a dodecamer of the complex composed of d-IIA^Glc^ and d-DctD_H216R_ ([d-IIA^Glc^/d-DctD_H216R_]_12_) showed insignificant ATPase activity, as [d-DctD_H216R_]_2_ and [d-IIA^Glc^/d-DctD_H216R_]_2_ did (black bars, Fig. 4B). It suggests that the apparent ATP hydrolysis by the fractions of [d-IIA^Glc^/DctD_D57Q_]_12_ was not derived from trace contamination of ATPase activity during GPC fractionation used in this study. Therefore, [d-IIA^Glc^/DctD_D57Q_]_12_, which has both DNA-binding affinity and ATPase activity, is a transcriptionally active form of d-DctD.

### [d-IIA^Glc^/DctD_D57Q_]_12_ specifically binds to the sequences homologous to a *Rhizobium* DctD-binding site

**Fig 5:**
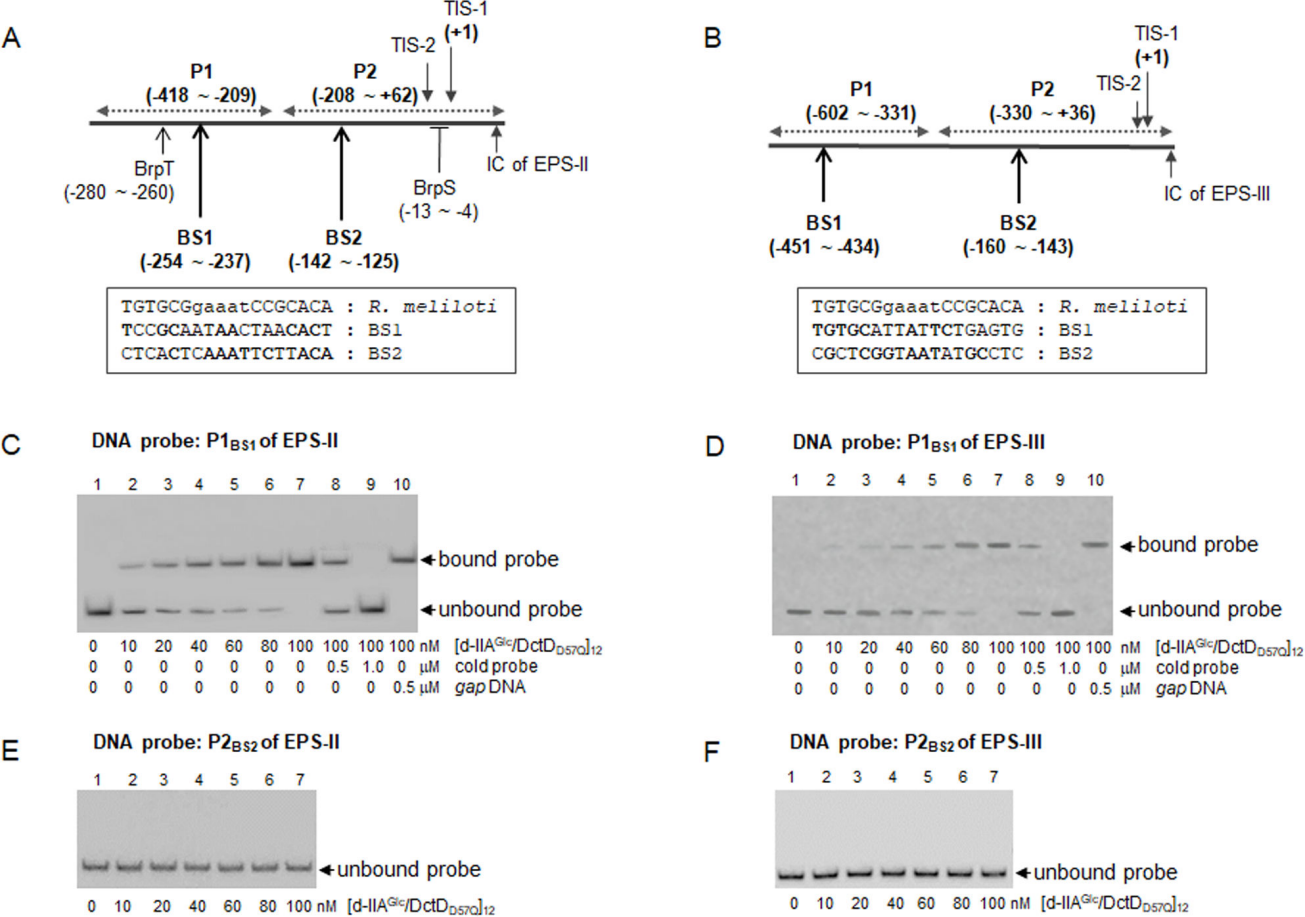
Localization of [d-IIA^Glc^/DctD_D57Q_]_12_-binding sites in the upstream regions of EPS-II and EPS-III clusters. (**A and B**) Two putative DctD-binding sites in the EPS-II and EPS-III clusters. Two sites (designated as BS1 for the remote site and BS2 for the close site from TIS-1) showing moderate identity to the DctD-binding sequences previously found in *R. meliloti* (5′-TGTGCGgaaatCCGCACA-3′; 15) were localized in the upstream regions of two gene clusters: the nucleotides positioned at −254 to −237 (BS1) and −142 to −125 (BS2) relative to RpoN-dependent TIS-1 of EPS-II (**A**) and the nucleotides positioned at −451 to −434 (BS1) and −160 to −143 (BS2) relative to the TIS-1 of EPS-III (**B**). Homologous nucleotides in each site were marked by boldfaces. In case of the EPS-II cluster (or *brp* operon), the regulatory sites interacting with the other transcription factors, such as BrpS and BrpT, were marked (16, 17). (**C–F**) Binding of [d-IIA^Glc^/DctD_D57Q_]_12_ to the probes containing either BS1 or BS2. DNA probes containing BS1 of EPS-II (**C**) or EPS-III (**D**), which were designated as P1_BS1_ (a 210 bp DNA fragment from −418 to −209 of EPS-II and a 272 bp DNA fragment from −602 to −331 of EPS-III), were prepared for EMSA. Similarly, the probes containing BS2 of EPS-II (**E**) or EPS-III (**F**) were prepared, which were designated as P2_BS2_ (a 270 bp DNA fragment from −208 to +62 of EPS-II and a 366 bp DNA fragment from −330 to +36 of EPS-III). Each probe was labeled and 100 nM was incubated with various concentrations of [d-IIA^Glc^/DctD_D57Q_]_12_ up to 100 nM. To verify the specific binding to P1_BS1_ (**C and D**), the identical but unlabeled DNA fragments (cold probes) and the noncompetitive *gap* DNA were included. The resultant reaction mixtures were subjected to 8% native polyacrylamide gel electrophoresis, and DNA bands corresponding to the unbound or bound probes were indicated by arrows. Lanes 1, probe only; lanes 2, probe with 10 nM of [d-IIA^Glc^/DctD_D57Q_]_12_; lanes 3, probe with 20 nM of [d-IIA^Glc^/DctD_D57Q_]_12_; lanes 4, probe with 40 nM of [d-IIA^Glc^/DctD_D57Q_]_12_; lanes 5, probe with 60 nM of [d-IIA^Glc^/DctD_D57Q_]_12_; lanes 6, probe with 80 nM of [d-IIA^Glc^/DctD_D57Q_]_12_; lanes 7, probe with 100 nM of [d-IIA^Glc^/DctD_D57Q_]_12_; lanes 8, probe with 100 nM of [d-IIA^Glc^/DctD_D57Q_]_12_ and 0.5 µM of cold probe; lanes 9, probe with 100 nM of [d-IIA^Glc^/DctD_D57Q_]_12_ and 1.0 µM of cold probe; and lanes 10, probe with 100 nM of [d-IIA^Glc^/DctD_D57Q_]_12_ and 0.5 µM of *gap* DNA.

It has been commonly observed that many members of the Group I bEBP bind to multiple sites in the upstream region of a target gene to activate its transcription (3). Two sites were localized in the upstream region of *dctA* of *Rhizobium* species (14). Additionally, the DctD-binding sequences, 5′-TGTGCGgaaatCCGCACA-3′, have been identified in *Rhizobium meliloti* (15). Using these sequences, the putative UASs of the EPS-II and EPS-III clusters in *V. vulnificus*, which are homologous to the *R. meliloti* DctD-binding sequences, were searched in their upstream regions. Two sites with homology to the consensus sequences were discernible, which were designated as BS1 remote from TIS-1 (−254 to −237 in EPS-II and −451 to −434 in EPS-III) and BS2 close to TIS-1 (−142 to −125 in EPS-II and −160 to −143 in EPS-III) (Fig. 5A and B). To determine whether [d-IIA^Glc^/DctD_D57Q_]_12_ could bind to these putative BSs, DNA probes containing a single BS of each gene cluster (P1_BS1_ for the probe including BS1 and P2_BS2_ for the probe including BS2) were prepared for electrophoretic mobility shift assay (EMSA). Only the probes containing BS1s were bound by [d-IIA^Glc^/DctD_D57Q_]_12_ and produced a shifted band in a concentration-dependent manner (Fig. 5C and D). Furthermore, the shifted bands were decreased by the addition of the cold probes but persisted by the addition of the noncompetitive *gap* DNA, indicating the specific binding of [d-IIA^Glc^/DctD_D57Q_]_12_ to the BS1-probes. In contrast, the probes containing BS2s did not produce any retarded band, even in the presence of the highest concentration of [d-IIA^Glc^/DctD_D57Q_]_12_ used in this study (up to 100 nM; Fig. 5E and F). These results indicate that [d-IIA^Glc^/DctD_D57Q_]_12_ efficiently binds to P1s containing the nucleotide sequences in BS1s.

### Interaction of [d-IIA^Glc^/DctD_D57Q_]_12_ to DNAs containing the specific binding sites is required for successful transcription of the target gene clusters

**Fig 6:**
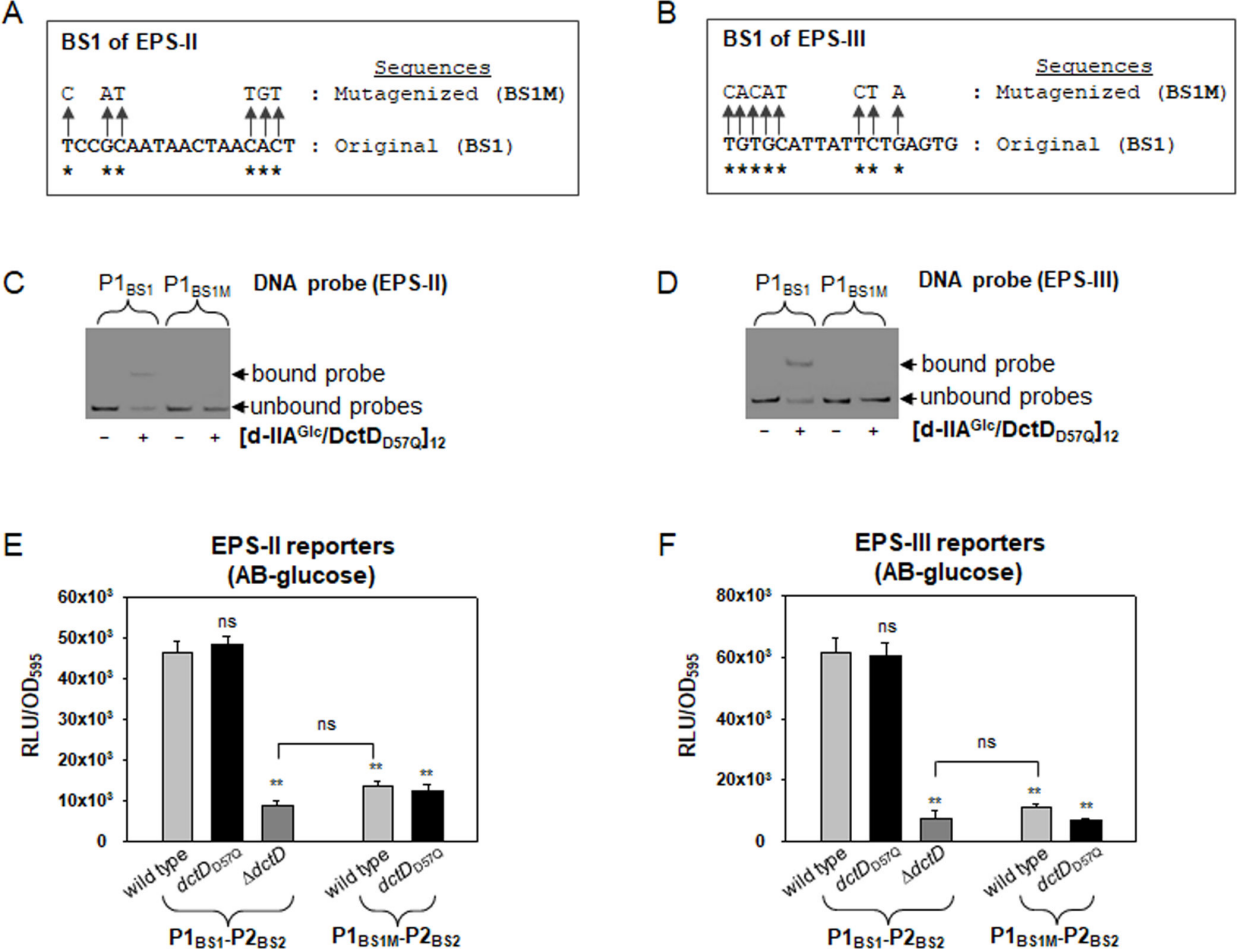
Effect of the mutations in the BS1s on binding by [d-IIA^Glc^/DctD_D57Q_]_12_ and transcription of the EPS clusters. (**A and B**) Mutagenesis of the BS1s of EPS-II and EPS-III clusters. The nucleotides in BS1s of EPS-II (**A**) and EPS-III (**B**), which are conserved in the *R. meliloti* DctD-binding consensus sequences (5′-TGTGCGgnnntCCGCACA-3′; 15), were marked with asterisks. These conserved nucleotides were substituted with other nucleotides, as indicated by arrows, to produce BS1M. (**C and D**) DNA-binding affinity of [d-IIA^Glc^/DctD_D57Q_]_12_ to the probes containing BS1M. DNA fragments of P1 containing BS1 (P1_BS1_) or BS1M (P1_BS1M_) were prepared from the EPS-II (**C**) and EPS-III (**D**) clusters. Then, EMSA was performed as described in Fig. 5. Labeled DNA bands corresponding to the unbound or bound probes, which were resolved in 6% native polyacrylamide gel, were indicated by arrows. (**E and F**) Expression of the BS1M-containing transcription reporters of EPS-II and EPS-III clusters. The *luxAB*-based transcription reporters fused with the original upstream regulatory regions (P1_BS1_-P2_BS2_; 18) or the mutagenized upstream regions (P1_BS1M_-P2_BS2_) of EPS-II (**E**) and EPS-III (**F**) were transferred to the wild type and *dctD*_D57Q_ (7). Then, *V. vulnificus* cells were grown in AB-glucose supplemented with tetracycline (3 µg/mL). At an OD_595nm_ of 0.4, aliquots of bacterial cells were harvested, and their luciferase activities were measured, as described in the Materials and Methods section. The expression of each reporter was presented as normalized values: the relative light unit (RLU) divided by the cell mass (OD_595nm_) of each sample. As a negative control, Δ*dctD* strain carrying the original reporter was included in each assay. *P-*values for comparison with the P1_BS1_-P2_BS2_ reporter in the wild type or Δ*dctD* were indicated (**P < 0.001; ns, not significant).

Next, to confirm whether the putative BS1s were directly and specifically recognized and bound by [d-IIA^Glc^/DctD_D57Q_]_12_, P1 probes containing the mutagenized BS1s were prepared by substituting the conserved nucleotides (BS1Ms; Fig. 6A and B). An EMSA using P1 probes containing mutagenized BS (P1_BS1M_) in the presence of [d-IIA^Glc^/DctD_D57Q_]_12_ did not show any retarded band (Fig. 6C and D). These results indicated that the specific binding of [d-IIA^Glc^/DctD_D57Q_]_12_ to P1 was achieved through its interaction with the regions containing the BS1s of the two gene clusters.

To determine whether BS1 is the actual *cis*-element determining the *in-vivo* transcriptional activation by [d-IIA^Glc^/DctD_D57Q_]_12_, *luxAB*-based transcription reporters fused with the original DNA fragments of the gene clusters (P1_BS1_-P2_BS2_; 18)or DNA fragments containing mutated BS1s (P1_BS1M_-P2_BS2_) were prepared. Their expression was monitored in wild-type and *dctD*_D57Q_ *V. vulnificus* grown in AB-glucose medium, in which IIA^Glc^ and DctD were present in dephosphorylated forms, as previously shown by Kang and Lee (7) (Fig. 6E and F). Compared to the original reporters of EPS-II and EPS-III clusters, the expression of P1_BS1M_-P2_BS2_-reporters was significantly impaired, which was almost at the same levels as those in Δ*dctD V. vulnificus*. These results indicate the critical role of BS1 in transcriptional activation by [d-IIA^Glc^/DctD_D57Q_]_12_.

### Specific nucleotides in the downstream regions of BS1s (BS1[dn]) are additionally required for successful binding of [d-IIA^Glc^/DctD_D57Q_]_12_

**Fig 7:**
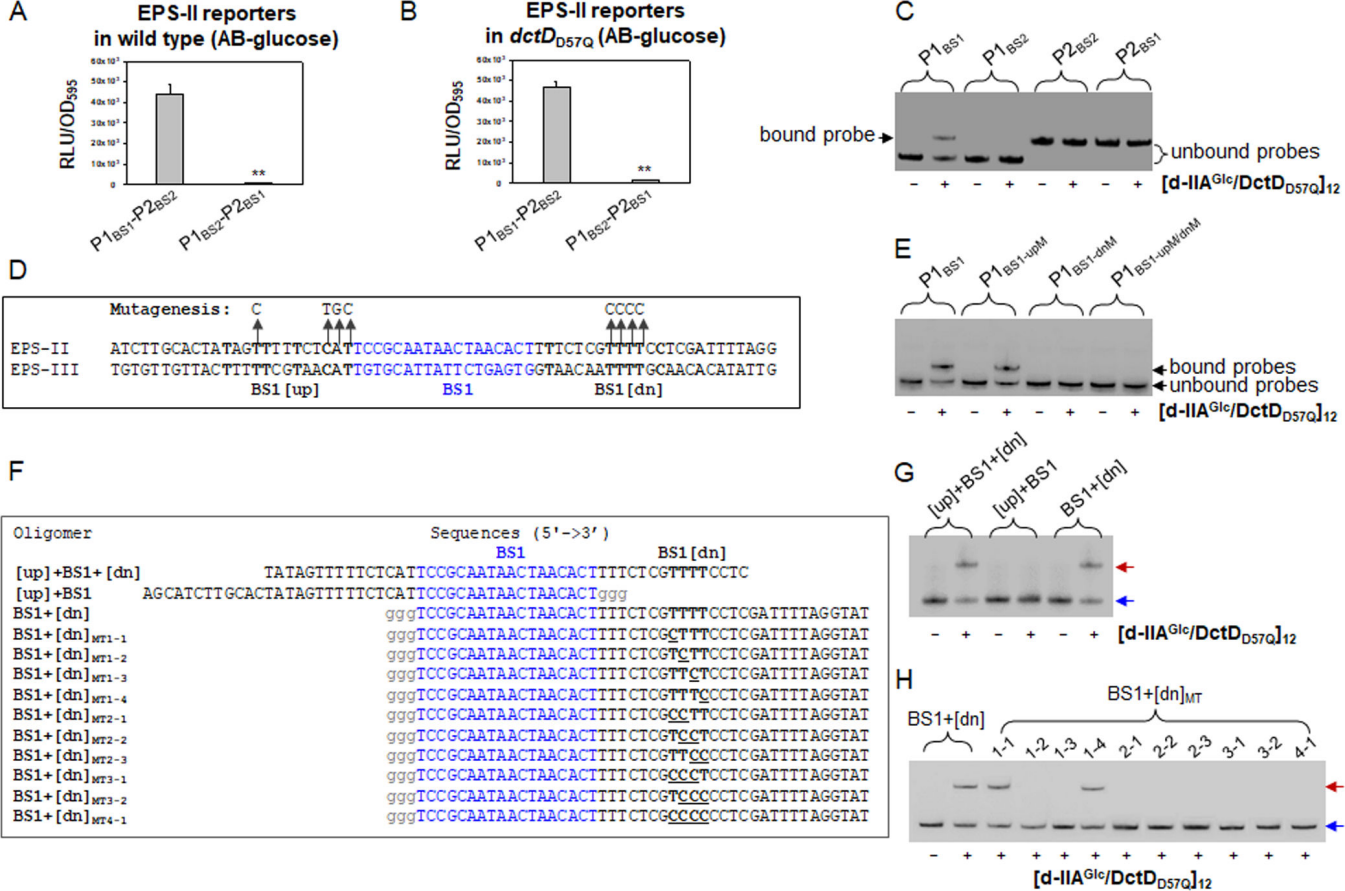
Characterization of the flanking region of BS1 for binding by [d-IIA^Glc^/DctD_D57Q_]_12_ and successful expression of EPS-II cluster. (**A and B**) Expression of the BS-switched transcription reporter of EPS-II. A mutagenized reporter was constructed by switching the positions of the original BS1 and BS2 of the EPS-II cluster, to produce P1_BS2_-P2_BS1_. Its expression in wild type (**A**) and *dctD*_D57Q_ (**B**) was compared to that of the original reporter (P1_BS1_-P2_BS2_). At an OD_595nm_ of 0.4 in AB-glucose supplemented with tetracycline (3 µg/mL), the expression of each reporter was estimated and presented with normalized values, RLU per OD_595nm_, as described in Fig. 6. *P-*values for comparison with the original reporter were indicated (**P < 0.001). (**C**) DNA-binding affinity of [d-IIA^Glc^ DctD_D57Q_]_12_ to BS-switched probes. Various DNA fragments of the EPS-II cluster, including the original P1 (P1_BS1_), switched P1 (P1_BS2_), the original P2 (P2_BS2_), and switched P2 (P2_BS1_), were prepared for EMSA using [d-IIA^Glc^/DctD_D57Q_]_12_. Each probe (100 nM) was mixed with 40 nM [d-IIA^Glc^/DctD_D57Q_]_12_. DNA bands corresponding to the unbound probes and the probe bound by [d-IIA^Glc^/DctD_D57Q_]_12_ were indicated by brackets and arrow, respectively. (**D**) Mutagenesis of BS1-flanking regions. The nucleotide sequences flanking the BS1s are conserved in the EPS-II and EPS-III clusters (marked with boldfaces). Nucleotides upstream of the EPS-II BS1 (BS1[up]) and downstream of the EPS-II BS1 (BS1[dn]) were mutagenized as indicated by arrows and substituted nucleotides, to produce BS1-dnM and BS1-upM, respectively. (**E**) DNA-binding affinity of [d-IIA^Glc^/DctD_D57Q_]_12_ to the probes containing BS1-dnM and BS1-upM. DNA fragments of the EPS-II cluster, including the original P1 (P1_BS1_), upstream-mutagenized P1 (P1_BS1-upM_), downstream-mutagenized P1 (P1_BS1-dnM_), and P1 mutagenized both upstream and downstream (P1_BS1-upM/dnM_), were prepared for EMSA using [d-IIA^Glc^/DctD_D57Q_]_12_. Each probe (100 nM) was mixed with 40 nM [d-IIA^Glc^/DctD_D57Q_]_12_, and resultant reaction mixtures were run on a native gel, as described above. (**F**) Preparation of various oligomers containing BS1. Nucleotide oligomers containing BS1 with both flanking regions ([up] + BS1 + [dn]), BS1 with upstream sequences only ([up] + BS1), and BS1 with downstream sequences only (BS1 + [dn]) were prepared. Series of BS1 + [dn] derivatives, BS1 + [dn]_MT_, whose Ts in BS1[dn] were mutagenized in a combinatorial way, were prepared. Eighteen nucleotides corresponding to the BS1 of the EPS-II cluster were blue-colored, and the extra three Gs linked to the 3’-end of [up] + BS1 and the 5’-end of BS1 + [dn] oligomers were gray-colored. Four nucleotides in BS1 + [dn] and its derivatives were bold-faced. (**G, H**) DNA-binding affinity of [d-IIA^Glc^/DctD_D57Q_]_12_ to various oligomers. Each oligomer (100 nM) was labeled and used for EMSA. Oligomers of [up] + BS1 + [dn], [up] + BS1, and BS1 + [dn] were mixed to reaction buffers without (−) and with (+) 40 nM [d-IIA^Glc^/DctD_D57Q_]_12_ (**G**). Ten mutant oligomers covering from BS1+[dn]_MT1-1_ to BS1+[dn]_MT4-1_ were mixed with [d-IIA^Glc^/DctD_D57Q_]_12_, and resultant reaction mixtures were subjected to 8% native polyacrylamide gel electrophoresis (lanes 3–12, **H**). As controls, BS1 + [dn] only (lane 1) and BS1 + [dn] with [d-IIA^Glc^/DctD_D57Q_]_12_ (lane 2) were included in the same gel. Oligomer bands corresponding to the unbound and bound probes were indicated by blue and red arrows, respectively.

Binding sequences for *R. meliloti* DctD, which comprise 18 nucleotides, have been derived from footprint assays using the truncated DctD mimicking phosphorylated DctD (p-DctD) (19). Considering the molecular size of [d-IIA^Glc^/DctD_D57Q_]_12_, it was plausible to speculate that this complex might interact with an extended region in the target DNA. Therefore, we further tested whether the DNA regions flanking BS1s were required for successful transcriptional activation by [d-IIA^Glc^/DctD_D57Q_]_12_. For this purpose, another *luxAB*-based transcription reporter of the EPS-II cluster was prepared using DNA fragment, in which the locations of BS1 and BS2 were switched to produce P1_BS2_-P2_BS1_. Then, its expression was monitored in wild-type (Fig. 7A) and *dctD*_D57Q_ (Fig. 7B) *V. vulnificus* grown in AB-glucose medium. Although a reporter having P1_BS2_-P2_BS1_ contained the intact BS1 sequences, its expression was not induced in both strains of *V. vulnificus*, compared to the original reporter having P1_BS1_-P2_BS2_.

To elucidate the reason for the lack of induction of the P1_BS2_-P2_BS1_ expression, two probes with switched BSs, P1_BS2_ and P2_BS1_, were prepared for EMSA in the presence of [d-IIA^Glc^/DctD_D57Q_]_12_. No binding of [d-IIA^Glc^/DctD_D57Q_]_12_ to P1_BS2_ or P2_BS1_ was observed (Fig. 7C). This suggests that in addition to the 18 nucleotide-long BS1, the extra nucleotide sequences in P1 were required for binding by [d-IIA^Glc^/DctD_D57Q_]_12_ and the successful activation of EPS-II cluster transcription.

Alignment of the nucleotide sequences flanking the BS1s of EPS-II and EPS-III revealed the presence of conserved nucleotides in the upstream (BS1[up]) and downstream (BS1[dn]) regions (Fig. 7D). To test whether these selected sequences could affect the binding of [d-IIA^Glc^/DctD_D57Q_]_12_, conserved nucleotides in each flanking region were mutagenized to produce P1 with mutagenized upstream (P1_BS1-upM_), mutagenized downstream (P1_BS1-dnM_), or mutagenized upstream and downstream (P1_BS1-upM/dnM_) (Fig. 7D). When each probe was treated with [d-IIA^Glc^/DctD_D57Q_]_12_, the probes mutagenized in the stretch of the four Ts located downstream of BS1 failed to be bound by [d-IIA^Glc^/DctD_D57Q_]_12_ (Fig. 7E), suggesting a critical role for BS1 and BS1[dn] in the interaction between P1 and [d-IIA^Glc^/DctD_D57Q_]_12_.

To verify that the sequences in BS1 and BS1[dn] containing multiple Ts were sufficient for [d-IIA^Glc^/DctD_D57Q_]_12_ to successfully bind to P1, three kinds of 27 nucleotide-long oligomers were prepared: [up] + BS1 + [dn], [up] + BS1, and BS1 + [dn] (Fig. 7F). An EMSA using these probes with [d-IIA^Glc^/DctD_D57Q_]_12_ revealed that oligonucleotides containing both BS1 and BS1[dn] were efficiently bound by [d-IIA^Glc^/DctD_D57Q_]_12_ (Fig. 7G). These results demonstrate that BS1, together with the downstream array of Ts, is required for binding by [d-IIA^Glc^/DctD_D57Q_]_12_.

### Alignments of BS1s of various DctD-regulons and localization of BS1[dn] for binding of [d-IIA^Glc^/DctD_D57Q_]_12_

**Fig 8:**
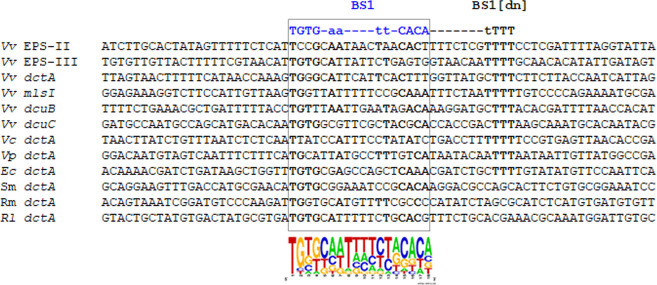
Proposed [d-IIA^Glc^/DctD_D57Q_]_12_-binding sequences in the DctD-regulons. The BS1 and BS1[dn] sequences of the EPS-II and EPS-III clusters were aligned with the putative BS1 and BS1[dn] in the upstream regions of *dctA* genes of *V. vulnificus* (Vv; 6), *Vibrio cholerae* (Vc; 21), *Vibrio parahaemolyticus* (Vp; VPY51_04360), *E. coli* (Ec; 20), *S. meliloti* (Sm; 14), *R. meliloti* (Rm; 15), and *R. leguminosarum* (Rl; 14). In addition, the upstream regions of the putative DctD-regulons, whose expression was assumed to be related to the metabolisms of dicarboxylic acids in *V. vulnificus*, were included in this analysis: malate synthase (*mlsI*, VVMO6_03414) and two C4-dicarboxylate transporters (*dcuB*, VVMO6_03835; *dcuC*, VVMO6_03887). Conserved regions among 12 BSs were marked by a dashed box and a nucleotide-frequency plot, and the highly conserved nucleotides among 12 BSs and 9 BS[dn]s were indicated with bold-faced letters.

To draw the consensus-binding sequences for [d-IIA^Glc^/DctD_D57Q_]_12_, putative BS1 and BS1[dn] were localized in the upstream regions of the known DctD-regulons (i.e., *dctA* genes of various bacterial species) and the tentative DctD-regulons of *V. vulnificus* (e.g., *mlsI*, *dcuB*, and *dcuC* genes). Alignment of these sequences with those of the EPS-II and EPS-III clusters revealed that 18 nucleotide-long BS1s are relatively well conserved as the following inverted repeat sequences: 5′-TGTG-aa----tt-CACA-3′ (Fig. 8). In addition, the arrays of multiple Ts apparently locate at the 7th or 8th nucleotide after the 3′-end of BS1s of the above genes encoded by three *Vibrio* species and *Escherichia coli* (6, 20, 21). In contrast, T-rich BS1[dn] was not discernible in the upstream regions of *dctA* genes of *Sinorhizobium meliloti* (14), *R. meliloti* (15), and *Rhizobium leguminosarum* (14): it is noteworthy that genes encoding the components of the typical glucose-PTS system are not present in *Rhizobium* and related species (22), suggesting the absence of [d-IIA^Glc^/d-DctD] complex in these bacteria.

The proposed BS1[dn]s contained three to five Ts, which led us to investigate the required number of Ts for binding by [d-IIA^Glc^/DctD_D57Q_]_12_. For this purpose, several oligonucleotides derived from the BS1 +[dn] of EPS-II cluster, whose Ts were sequentially mutated to produce a series of BS1 + [dn]_MT_ as described in Fig. 7F. EMSA using these probes, from BS1 + [dn]_MT1-1_ to BS1 + [dn]_MT4-1_, showed that the mutant probes containing at least three consecutive Ts, such as BS1 + [dn]_MT1-1_ and BS1 + [dn]_MT1-4_, were able to be bound by [d-IIA^Glc^/DctD_D57Q_]_12_ (Fig. 7H). Therefore, 18 nucleotide-long BS1 and 3 consecutive Ts in BS1[dn] are essential for binding by [d-IIA^Glc^/d-DctD]_12_.

## DISCUSSION

DctD is a well-known transcription factor belonging to Group I of bEBP, whose regulatory competence in activating RpoN-initiated transcription (23) is achieved after it is phosphorylated by its cognate sensor kinase, DctB. Interestingly, the transcriptionally inactive state of dephosphorylated DctD (d-DctD) is converted to its transcriptionally active form when it produces a complex with d-IIA^Glc^ (7). To date, transcriptional activation by dephosphorylated forms of other Group I bEBPs has not been reported. Our results suggest that d-DctD or the d-IIA^Glc^/d-DctD complex may have unique features that have not been observed in the well-studied members of Group I bEBPs. Therefore, to understand this phosphorylation-independent regulatory mechanism, the molecular characteristics of both d-DctD and the d-IIA^Glc^/d-DctD complex were investigated in this study using the DctD_D57Q_ and the DctD-regulons identified in *V. vulnificus* (6).

DctD_D57Q_, a form of recombinant DctD that mimics dephosphorylated DctD (7), exists in a dimeric conformation (Fig. 1). Although dimeric forms of dephosphorylated Group I bEBPs, such as NtrC and ZraR, were at least able to bind to specific DNA sequences (9, 24), dimeric d-DctD ([DctD_D57Q_]_2_) did not show any DNA-binding activity (Fig. 1C). Thus, while a dimeric form of truncated DctD has been shown to have DNA-binding affinity (13, 25), it is noteworthy that this recombinant protein was deleted in its regulatory domain and considered to be a constitutively active form.

DctD_D57Q_ specifically interacted with the recombinant protein of d-IIA^Glc^, which has been purified from cells grown under glucose-enriched conditions (7, 26), and formed a complex in a 1:1 molar ratio (Fig. 2D, 3B and D). This complex was present in either dimeric ([d-IIA^Glc^/DctD_D57Q_]_2_) or dodecameric ([d-IIA^Glc^/DctD_D57Q_]_12_) conformations (Fig. 2A), among which [d-IIA^Glc^/DctD_D57Q_]_12_ showed the ability to bind to target DNA and hydrolyze ATP molecules (Fig. 4).

It is generally considered that the oligomerization process of bEBPs is initiated by the dimerization of two monomers and followed by the formation of a hexamer via direct interaction among three dimers (3). However, a process deviated from the above stepwise oligomerization has been observed in PspF belonging to the Group IV bEBP, which lacks the regulatory domain and requires other proteins to control its ATPase activity and DNA-binding affinity (27). When DctD_D57Q_ was mixed with d-IIA^Glc^, a hexameric form of the complex was not detected. Thus, it is speculated that [d-IIA^Glc^/DctD_D57Q_]_2_ undergoes hexamerization to produce [d-IIA^Glc^/DctD_D57Q_]_12_, in which their ATPase domains can be positioned to attain the ATP hydrolyzing activity. The amino acid residues of DctD and IIA^Glc^ that are involved in the interaction between the two proteins have not yet been identified, and the structural characterization of this complex waits future study. Nonetheless, it has been postulated that d-IIA^Glc^ would interact with the regulatory domain of DctD to mimic the transcriptionally active p-DctD since structural modification of the phosphorylated regulatory domains is critically important for eliciting the ATPase activity of Group I bEBPs (12). Therefore, it is plausible that the arrangement of a dodecamer composed of 12 d-DctDs, to which regulatory domains are adhered by d-IIA^Glc^, should be different from the arrangements of a higher-order multimer of p-DctD.

The previous studies with the *Rhizobium* DctD showed that DctD-binding nucleotide sequences were 5′-TGTGCGgnnntCCGCACA-3′ and the multiple (i.e., at least two) binding sites were localized in the regions upstream *dctA* genes (14, 19, 28). . These findings led us to putatively localize multiple DctD-binding sites, which were homologous to the above sequences, in the upstream regions of the DctD-regulon, such as the EPS-II and EPS-III clusters. Among the two candidate binding sites, BS1 and BS2 (Fig. 5A and B), BS1s locating relatively far from the RpoN-initiated TISs were specifically bound by [d-IIA^Glc^/d-DctD]_12_ (Fig. 5C through F). Alignment of the putative BS1s in the upstream regions of various DctD-regulons showed the presence of relatively well-conserved inverted repeat sequences of 5′-TGTG-aa----tt-CACA-3′ (Fig. 8).

Structural studies using recombinant NtrC, a representative member of the Group I bEBP, suggest that each monomer in a dimer is directly involved in specifically binding the target DNA sequences consisting of 17 nucleotides (29). Therefore, the binding regions for [d-IIA^Glc^/DctD_D57Q_]_12_ may be wider than those determined using a truncated DctD dimer (14, 19, 28). To test this hypothesis, regions flanking BS1 were examined for their involvement in specific interactions with [d-IIA^Glc^/DctD_D57Q_]_12_. EMSA using various DNA probes, as shown in Fig. 7D and F, clearly demonstrated that both BS1 and its downstream region (BS1[dn]), containing at least three consecutive Ts, were essential for the *in vitro* binding of [d-IIA^Glc^/DctD_D57Q_]_12_ (Fig. 7E, G and H) and the *in vivo* transcriptional activation (Fig. 7A and B).

Cellular levels of the dephosphorylated forms of glucose-PTS components are highly increased in bacteria growing in the presence of glucose (30). Thus, cellular levels of [d-IIA^Glc^/d-DctD]_12_ and its transcriptional activity are mainly determined by glucose in the ambient environments. However, complex formation between d-DctD and d-IIA^Glc^ is further regulated by other carbon sources, such as glycerol (7). Glycerol kinase, GlpK, has high affinity to d-IIA^Glc^ (31, 32); thus, its presence results in decreased levels of cellular [d-IIA^Glc^/d-DctD]_12_ due to competition between GlpK and d-DctD for binding d-IIA^Glc^. Since d-IIA^Glc^ is a versatile protein capable of interacting with the other kinases of non-PTS sugars, including maltose, arabinose, melibiose, and raffinose (33), [d-IIA^Glc^/d-DctD]_12_-mediated regulatory pathways should be under the multilayered control of diverse carbon sources. Furthermore, complex formation with IIA^Glc^ may play an additional role in controlling d-DctD protein stability *in vivo*. In this case, the formation of [d-IIA^Glc^/d-DctD]_12_ could be a way modulating the appropriate cellular levels of total DctD.

Taken together, DctD, a response regulator comprising a two-component regulatory system, can activate transcription via an alternative pathway that is independent of its cognate sensor kinase, but dependent upon a dephosphorylated component of the glucose-PTS, IIA^Glc^. When the dodecamer of the d-IIA^Glc^/d-DctD complex is formed, it specifically binds to a single but extended BS and activates transcription of the target genes.

## MATERIALS AND METHODS

### Bacterial strains and culture conditions

Strains and plasmids used in this study are listed in Table S1. *V. vulnificus* strains were grown at 30°C in AB medium (300 mM NaCl, 50 mM MgSO_4_, 0.2% casamino acids, 10 mM potassium phosphate, 100 mM sodium phosphate, 1 mM L-arginine, pH7.5) (34) supplemented with glucose (33.4 mM). *E. coli* strains used for plasmid DNA preparation and conjugational transfer were grown at 37°C in Luria-Bertani (LB) medium (1% tryptone, 0.5% yeast extract, and 1% NaCl) (35). Antibiotics were added to AB or LB media at the following concentrations: for *V. vulnificus,* tetracycline at 3 µg/mL; and for *E. coli*, ampicillin at 100 µg/mL and tetracycline at 15 µg/mL.

### Purification and gel permeation chromatography of recombinant proteins

For the preparation of d-DctD_2_, pQE30-*dctD*_D57Q_ (7) was expressed in *E. coli* JM109 in the presence of 1 mM isopropyl β-d-thiogalactopyranoside. To prepare d-IIA^Glc^, pQE30-*crr* was expressed in the presence of glucose, as previously described (36). Each recombinant protein was purified using an Ni^+^-nitrilotriacetic acid affinity column (Bio-Rad). Next, 500 µL of the recombinant proteins of DctD_D57Q_, d-IIA^Glc^ and the d-IIA^Glc^/DctD_D57Q_ complex dissolved in a buffer (50 mM Tris-HCl [pH 8.0], 20 mM KCl, 50 mM MgCl_2_, and 100 mM NaCl) were applied to the AKTA-FPLC system (Amersham Biosciences) equipped with a Superdex 200 Increase 10/300 Gl column (GE Healthcare) (37). Each sample was fractionated using a running buffer (50 mM Tris-HCl [pH 8.0], 20 mM KCl, 50 mM MgCl_2_, and 300 mM NaCl) at a flow rate of 0.4 mL/min. The void volume of the column used in this assay was 7.2 mL, which is consistent with the previously reported value (38). The apparent molecular weights of the oligomeric proteins in the collected fractions were determined using an elution profile derived from the standard proteins (Protein Standard Mix 15–600 kDa, Sigma-Aldrich) as described in Fig. S1.

### Site-directed mutagenesis

DctD-binding sites (BS1) in the upstream regions of the EPS-II and EPS-III clusters were mutagenized using the overlap extension method (39) with the following sets of primers carrying the desirably substituted nucleotides: BS1M_II-F and BS1M_II-R for BS1 of EPS-II and BS1M_III-F and BS1M_III-R for BS1 of EPS-III (Table S2). To switch the positions of the BSs of EPS-II, the primer sets of EPS_II_BS1toBS2-F and EPS_II_BS1toBS2-R or EPS_II_BS2toBS1-F and EPS_II_BS2toBS1-R were used to produce P1_BS2_ or P2_BS1_, respectively. To mutagenize the nucleotide sequences in the upstream and downstream regions of BS1 of EPS-II, the primer sets of EPS_II_upst-F and EPS_II_upst-R or EPS_II_dnst_F and EPS_II_dnst_R were used to produce P1_BS1-upM_ or P1_BS1-dnM_, respectively. The resultant DNA fragments were cloned into pBlunt-TOPO (MGmed) and the mutagenized nucleotide sequences were verified by DNA sequencing. To construct pQE30-*dctD*_H216R_, the internal primers including the altered nucleotides for the 216th arginine of *V. vulnificus* DctD, H216R-F and H216R-R (13), were utilized as described above (39, Table S2). The resultant mutagenized *dctD* fragment was digested with BamHI and KpnI and then ligated to pQE30 as previously described (6).

### Electrophoretic mobility shift assay

EMSAs were performed with various kinds of probes containing a single BS or both BSs: DNA probes containing a single BS of EPS-II cluster [P1_BS1_ with BS1 (209 bp) and P2_BS2_ with BS2 (271 bp)] or EPS-III cluster [P1_BS1_ with BS1 (271 bp) and P2_BS2_ with BS2 (367 bp)]; a DNA probe containing both BSs of EPS-II cluster [P1_BS1_-P2_BS2_ (480 bp)]; and DNA probes containing switched BS of EPS-II cluster [P1_BS2_ (209 bp) and P2_BS1_ (271 bp)]. To analyze the flanking regions of BS1, oligonucleotides [up] + BS1 + [dn], [up] + BS1, BS1 + [dn], and mutant derivatives of BS1 + [dn] were prepared as described in Fig. 7F. DNA fragments were labeled with [γ-^32^P] ATP using T4 polynucleotide kinase (TaKaRa), and the resultant labeled probes were incubated in a reaction buffer (50 mM Tris-HCl [pH 8.0], 20 mM KCl, 50 mM MgCl_2_, and 100 mM NaCl) with various concentrations of recombinant proteins of DctD_D57Q_ (7) and d-IIA^Glc^ (36). The reaction mixtures were resolved on 6% or 8% native polyacrylamide gels.

### Measurement of ATPase activity

The enzymatic activity for ATP hydrolysis was measured by quantifying the phosphate released from ATP using ATPase/GTPase Activity Kit (Sigma-Aldrich). For the assays, FPLC fractions representing the specific multimeric states of DctD_D57Q_ and the d-IIA^Glc^/DctD_D57Q_ complex were diluted to 10 µmole/l with assay buffer (20 mM HEPES [pH 7.0], 150 mM NaCl, 5% glycerol, and 5 mM MgCl_2_) and mixed with 5 mM ATP. After the reaction mixtures (80 µL) were incubated at 37°C for 10 min, the mixtures were added with malachite green reagent (20 µL) and subjected to spectrophotometry at 620 nm (11). As a control, an ATPase-deficient DctD, DctD_H216R_, was purified from *E. coli* JM109 carrying pQE30-*dctD*_H216R_ after treated with alkaline-phosphatase (AP) as described (40). Then, dephosphorylated form of DctD_D216R_ (d-DctD_D216R_) and d-IIA^Glc^ complex was dissolved in a buffer (50 mM Tris-HCl [pH 8.0], 20 mM KCl, 50 mM MgCl_2_, and 100 mM NaCl) and applied to the AKTA-FPLC system equipped with a Superdex 200 Increase 10/300 Gl column. Fractionated dimeric d-DctD_H216R_ and d-IIA^Glc^/d-DctD_H216R_ complexes were used for ATPase assay.

### Measurement of transcriptional reporter plasmids fused with *luxAB* genes

The expression of various *luxAB*-based transcription reporters was monitored in *V. vulnificus* strains growing in AB-glucose medium, as previously described (6). At the designated time points, the bacterial culture aliquots were mixed with a luciferase substrate, *n*-decyl aldehyde (0.006%), and then the resultant light production was measured in a luminometer (GloMax 20/20 luminometer, Promega). Specific bioluminescence was presented by normalizing the RLU with respect to cell mass (OD_595_), as described (41).

### Statistical analyses

Results are expressed as means ± standard deviations of data from at least three independent experiments. Statistical analysis was performed using Student’s *t*-test (Systat Program, SigmaPlot version 9; Systat Software, Inc.). *P*-values are presented by one asterisk (^*^) or two asterisks (^**^) when 0.001 < *P* < 0.01 or *P* < 0.001, respectively.

## SUPPLEMENTAL MATERIAL

Figure S1 mbio.00330-24-s0001.pdf Gel permeation chromatography of standard proteins.

Figure S2 mbio.00330-24-s0002.pdf PAGE analysis of the mixture of d-IIA^Glc^ and DctD_D57Q_.

Table S1 mbio.00330-24-s0003.pdf Strains and plasmids used in this study.

Table S2 mbio.00330-24-s0004.pdf Oligonucleotides used in this study.

ASM does not own the copyrights to Supplemental Material that may be linked to, or accessed through, an article. The authors have granted ASM a non-exclusive, world-wide license to publish the Supplemental Material files. Please contact the corresponding author directly for reuse.
