# Supplementary material for: Characterization of the transcriptionally active form of dephosphorylated DctD complexed with dephospho-IIAGlc
Source: mBio. 2024 Apr 2;15(5):e00330-24. doi: 10.1128/mbio.00330-24 (PMC11077940; doi:10.1128/mbio.00330-24)
Supplement: Figure S1 — Gel permeation chromatography of standard proteins. [file mbio.00330-24-s0001.pdf]

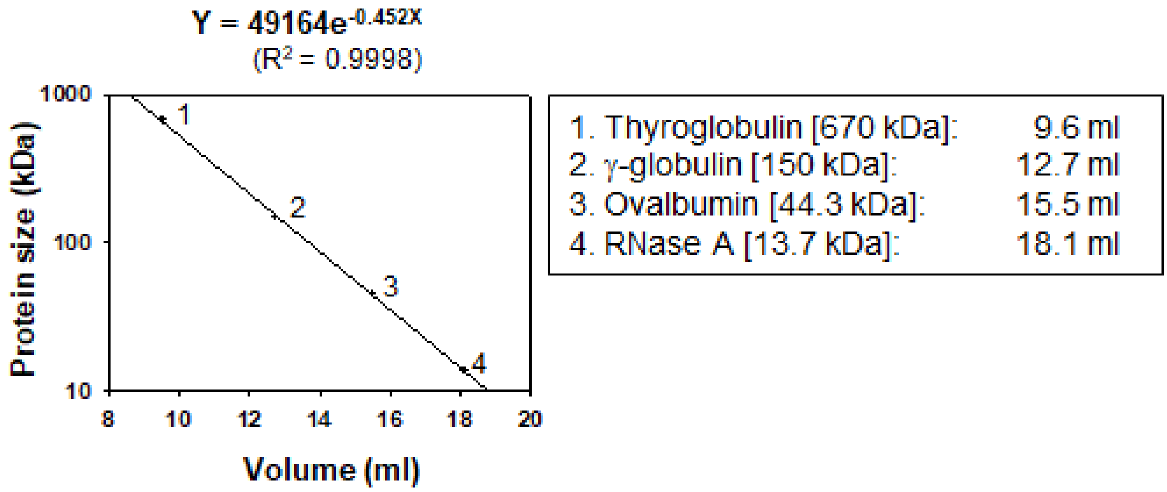

Figure S1. Gel permeation chromatography of standard proteins

A standard protein mixture (Protein Standard Mix 15-600 kDa, Sigma-Aldrich) containing thyroglobulin (670 kDa; 1), γ-globulin (150 kDa; 2), ovalbumin (44.3 kDa; 3), and RNase A (13.7 kDa; 4) was eluted through the same GPC as described in Fig. 1A. A plot of elution volume (X-axis) *vs* molecular weight (kDa) (Y-axis) was drawn, and a regression equation, $Y = 49164e^{-0.452X}$ (R^2 = 0.9998) was obtained. Using this equation, the eluted volumes of the peaks of $DctD_{D57Q}$ (Fig. 1A) and [d-IIA^{Glc}/$DctD_{D57Q}$] complexes (Fig. 2A) were converted to the corresponding molecular weight.
