# Supplementary material for: Characterization of the transcriptionally active form of dephosphorylated DctD complexed with dephospho-IIAGlc
Source: mBio. 2024 Apr 2;15(5):e00330-24. doi: 10.1128/mbio.00330-24 (PMC11077940; doi:10.1128/mbio.00330-24)
Supplement: Figure S2 — PAGE analysis of the mixture of d-IIAGlc and DctDD57Q. [file mbio.00330-24-s0002.pdf]

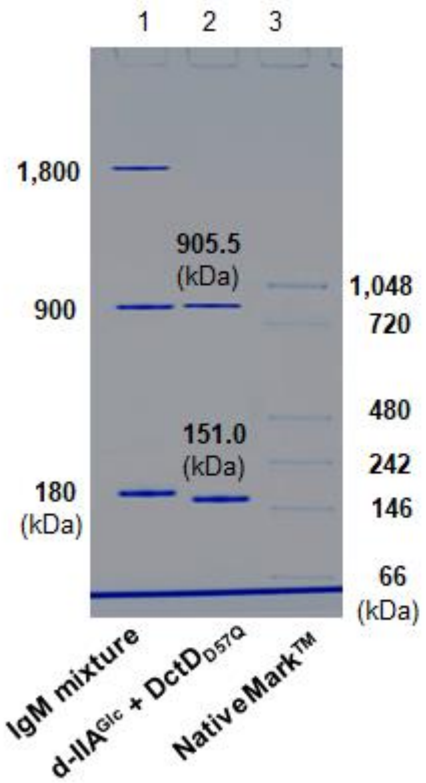

Figure S2. PAGE analysis of the mixture of d-IIA^{Glc} and DctD$_{D57Q}$.

A mixture containing two recombinant proteins, d-IIA^{Glc} and DctD$_{D57Q}$, was run in an 8-16% gradient polyacrylamide gel (Tris-Glycine-PAG SDS Gel, LABISKOMA). Two different protein standards were included in the same gel: one contained di-pentameric IgM (1,800 kDa), pentameric IgM (900 kDa), and monomeric IgM (180 kDa) [IgM from Human Serum, Sigma-Aldrich; lane 1]; and the other standard contained pentameric IgM tagged with GST (1,048 kDa), apoferritin band 1 (720 kDa), apoferritin band 2 (480 kDa), β-phycoerythrin (242 kDa), lactate dehydrogenase (146 kDa), and bovine serum albumin (66 kDa) [NativeMark™ Unstained Protein Standard, Thermo Fisher Scientific; lane 3]. The sizes of the [d-IIA^{Glc}/DctD$_{D57Q}$] complexes, which were estimated by GPC analysis (Fig. 2A), were indicated above the corresponding bands (lane 2).
