# Supplementary material for: Characterization of the transcriptionally active form of dephosphorylated DctD complexed with dephospho-IIAGlc
Source: mBio. 2024 Apr 2;15(5):e00330-24. doi: 10.1128/mbio.00330-24 (PMC11077940; doi:10.1128/mbio.00330-24)
Supplement: Table S1 — Strains and plasmids used in this study. [file mbio.00330-24-s0003.pdf]

Table S1. Strains and plasmids used in this study

Strain/Plasmid	Genotype	Reference [a]
V. vulnificus		
MO6-24/O	Clinical isolate	(1)
Δ*dctD*	MO6-24/O, Δ*dctD*	(2)
dctD$_{\mathrm{D57Q}}$	MO6-24/O, *dctD*$_{2}$ with 57th amino acid substituted	(3)
E. coli		
DH5α	f80d*lacZ* DM15 *recA1 endA1 gyrA96 relA1 thi-1*	Laboratory collection
	hsdR17(r$_{\mathrm{K}}^{-}$ m$_{\mathrm{K}}^{-}$) *supE44 deoR Δ*(*lacZYA-argF*) *U169*	
SM10λ*pir*	*thi-1 thr leu tonA lacY supE recA*::<br>Rp4-2-Tc::Mu*λpir,* OriT of RP4, Km^{R}	(4)
JM109	*endA1 recA1 gyrA96 thi-1 hsdR17*(r$_{\mathrm{K}}^{-}$ m$_{\mathrm{K}}^{-}$) *relA*1	Promega
	supE44 (*lac-proAB*)[F'*traD3 6proAB lacI*^{q}*Z* M15]	
**Plasmids**		
pBlunt-TOPO	A cloning vector, Km^{R}, Ap^{R}	MGmed
pT-EII-P1 [BS1]	pBlunt-TOPO carrying 209-bp P1 including BS1 of EPS-II	This study
pT-EII-P2 [BS2]	pBlunt-TOPO carrying 271-bp P2 including BS2 of EPS-II	This study
pT-EII-P1 [BS1M]	pT-EII-P1 [BS1], but mutated BS1	This study
pT-EII-P1 [BS2]	pT-EII-P1 [BS1], but substituted with BS2 at BS1 position	This study
pT-EII-P2 [BS1]	pT-EII-P2 [BS2], but substituted with BS1 at BS2 position	This study
pT-EII	pBlunt-TOPO carrying 480-bp P1+P2 of EPS-II	(5)
	(having P1$_{\mathrm{BS1}}$+P2$_{\mathrm{BS2}}$, previously pTOP-EPS-II)	
pT-EII [BS1M]	pT-EII, but mutated BS1 (having P1$_{\mathrm{BS1M}}$+P2$_{\mathrm{BS2}}$)	This study
pT-EII [P1$_{\mathrm{BS2}}$-P2$_{\mathrm{BS1}}$]	pT-EII having P1$_{\mathrm{BS2}}$+P2$_{\mathrm{BS1}}$	This study
pT-EII-P1 [upM]	pT-EII-P1 [BS1] with mutations in upstream of BS1	This study
pT-EII-P1 [dnM]	pT-EII-P1 [BS1] with mutations in downstream of BS1	This study
pT-EII-P1 [upM/dnM]	pT-EII-P1 [BS1] with mutations in up- and down-streams of BS1	This study
pT-EIII-P1 [BS1]	pBlunt-TOPO carrying 271-bp P1 including BS1 of EPS-III	This study
pT-EIII-P2 [BS2]	pBlunt-TOPO carrying 367-bp P2 including BS2 of EPS-III	This study
pT-EIII-P1 [BS1M]	pT-EIII-P1 [BS1], but mutated BS1	This study
pT-EIII	pBlunt-TOPO carrying 638-bp P1+P2 of EPS-III	(5)
	(having P1$_{\mathrm{BS1}}$+P2$_{\mathrm{BS2}}$, previously pTOP-EPS-III)	
pT-EIII [BS1M]	pT-EIII, but mutated BS1 (having P1$_{\mathrm{BS1M}}$+P2$_{\mathrm{BS2}}$)	This study
pHK0011	pRK415 with promoterless *luxAB*, Tc^{R}	(6)

pCB012	EPS-II cluster::*luxAB* transcriptional fusion in pHK0011	(5)
pSB-EII [BS1M]	pCB012, but mutated BS1 (having $P1_{BS1M}$+$P2_{BS2}$)	This study
pSB-EII [$P1_{BS2}$-$P2_{BS1}$]	pCB012 having $P1_{BS2}$+$P2_{BS1}$	This study
pCB013	EPS-III cluster::*luxAB* transcriptional fusion in pHK0011	(5)
pSB-EIII [BS1M]	pCB013, but mutated BS1 (having $P1_{BS1M}$+$P2_{BS2}$)	This study
pQE30	Expression vector, Ap^R	Qiagen
pQE30-*dctD*$_{D57Q}$	pQE30 containing *dctD*$_{D57Q}$	(3)
pQE30-*dctD*$_{H216R}$	pQE30 containing *dctD*$_{H216R}$	This study
pQE30-*crr*	pQE30 containing 522-bp original *crr* ORF	(7)

[a] **References**

1. Wright AC, Simpson LM, Oliver JD, Morris JG Jr. 1990. Phenotypic evaluation of capsular transposon mutants of *Vibrio vulnificus*. *Infect Immun* 58:1769-1773.

2. Kang S, Park H, Lee KJ, Lee KH. 2021. Transcription activation of two clusters for exopolysaccharide biosynthesis by phosphorylated DctD in *Vibrio vulnificus*. *Environ Microbiol* 23:5364-5377.

3. Kang S, Lee KH. 2022. Transition of dephospho-DctD to the transcriptionally active state via interaction with dephospho-IIA^{Glc}. *mBio* 13: e03839-21.

4. Simon R, Priefer U, Pühler A. 1983. A broad host range mobilization system for *in vivo* genetic engineering: transposon mutagenesis in gram negative bacteria. *Nat Biotechnol* 1:784-791.

5. Kim HS, Park SJ, Lee KH. 2009. Role of NtrC-regulated exopolysaccharides in the biofilm formation and pathogenic interaction of *Vibrio vulnificus*. *Mol Microbiol* 74:436-453.

6. Jeong HS, Jeong KC, Choi HK, Park KJ, Lee KH, Rhee JH, Choi SH. 2001. Differential expression of *Vibrio vulnificus* elastase gene in a growth phase-dependent manner by two different types of promoters. *J Biol Chem* 27:13875-13880.

7. Lee KJ, Jeong CS, An YJ, Lee HJ, Park SJ, Seok YJ, Kim P, Lee JH, Lee KH, Cha SS. 2011. FrsA functions as a cofactor-independent decarboxylase to control metabolic flux. *Nat Chem Biol* 7:434-436.
