# Supplementary material for: Characterization of the transcriptionally active form of dephosphorylated DctD complexed with dephospho-IIAGlc
Source: mBio. 2024 Apr 2;15(5):e00330-24. doi: 10.1128/mbio.00330-24 (PMC11077940; doi:10.1128/mbio.00330-24)
Supplement: Table S2 — Oligonucleotides used in this study. [file mbio.00330-24-s0004.pdf]

Table S2. Oligonucleotides used in this study

Probe/Protein	Primer name	Sequences [a] (5'->3')
P1$_{BS1}$	EPS_II_P1-R	CCTAAATCATAAAAATCTAATACCTAAAATCG
P1$_{BS1M}$	BS1M_II-F	CTCAT*C*CC*AT*AATAACTAA*TGT*TTTTCTCG
	BS1M_II-R	CGAGAAAA*ACA*TTAGTTATT*AT*GG*G*ATGAG
P2$_{BS2}$	EPS_II_P2-F	CGATTTTAGGTATTAGATTTTTATGATTTAGG
P1$_{BS2}$	EPS_II_BS1toBS2-F	GTTTTTCTCAT*CTCACTCAAATTCTTACA*TTTCTCG
	EPS_II_BS1toBS2-R	CGAGAAA*TGTAAGAATTTGAGTGAG*ATGAGAAAAAC
P2$_{BS1}$	EPS_II_BS2toBS1-F	CCACTAACCAAA*TCCGCAATAACTAACACT*CTTCCTTGC
	EPS_II_BS2toBS1-R	GCAAGGAAG*AGTGTTAGTTATTGCGGA*TTTGGTTAGTGG
P1$_{BS1\text{-}upM}$	EPS_II_upst-F	CACTATAGTTTTTCT*TGC*TCCGCAATAAC
	EPS_II_upst_R	GTTATTGCGGA*GCA*AGAAAAACTATAGTG
P1$_{BS1\text{-}dnM}$	EPS_II_dnst_F	TAACACTTTTCTCG*CCCC*CCTCGATTTTAGGT
	EPS_II_dnst_R	ACCTAAAATCGAGG*GGGG*CGAGAAAAGTGTTA
[up]+BS1+[dn]	EPS_II_BS1_1-18_F	TATAGTTTTTCTCATTCCGCAATAACTAACACTTTTCTCGTTTTCCTC
	EPS_II_BS1_1-18_R	GAGGAAAACGAGAAAAGTGTTAGTTATTGCGGAATGAGAAAAACTATA
[up]+BS1	EPS_II_BS1_up-F	AGCATCTTGCACTATAGTTTTTCTCATTCCGCAATAACTAACACT*GGG*
	EPS_II_BS1_up-R	*CCC*AGTGTTAGTTATTGCGGAATGAGAAAAACTATAGTGCAAGATGCT
BS1+[dn]	EPS_II_BS1_dn-F	*GGG*TCCGCAATAACTAACACTTTTCTCGTTTTCCTCGATTTTAGGTAT
	EPS_II_BS1_dn-R	ATACCTAAAATCGAGGAAAACGAGAAAAGTGTTAGTTATTGCGGA*CCC*
P1$_{BS1}$	EPS_III_P1-R	CCAGCACGCCGACCCACAATAACAAGGCGTTGGGC
P1$_{BS1M}$	BS1M_III-F	CGTAACAT*CACAT*ATTAT*CT*T*A*AGTGGTAAC
	BS1M_III-R	GTTACCACT*TAAG*ATAAT*ATGTG*ATGTTACG
P2$_{BS2}$	EPS_III_P2-F	GCCCAACGCCTTGTTATTGTGGGTCGGCGTGCTGG
DctD$_{H216R}$	H216R-F	GAGCTATTTGGTC*G*TGAGGCCGGGGCG
	H216R-R	CGCCCCGGCCTCA*C*GACCAAATAGCTC
	DctD-F	GGGGATCCATGGACAAGTGGTATTTCTCGATGGATGCAGTTTCTTT [b]
	DctD-R	GGGGTACCTCAGCTGTCGGTGTAATCGGCGCGATTCAGACC [b]

[a] Altered nucleotides for mutagenizing are indicated with italicized letters and their usages in cloning experiments are described in "Materials and Methods".

[b] Restriction sites are underlined, as previously described (Kang S, Park H, Lee KJ, Lee KH. 2021. Transcription activation of two clusters for exopolysaccharide biosynthesis by phosphorylated DctD in *Vibrio vulnificus*. *Environ Microbiol* 23:5364-5377.).
